# A bioinformatics investigation into molecular mechanism of Yinzhihuang granules for treating hepatitis B by network pharmacology and molecular docking verification

**DOI:** 10.1038/s41598-020-68224-7

**Published:** 2020-07-10

**Authors:** Jingyuan Zhang, Xinkui Liu, Wei Zhou, Guoliang Cheng, Jiarui Wu, Siyu Guo, Shanshan Jia, Yingying Liu, Bingbing Li, Xiaomeng Zhang, Miaomiao Wang

**Affiliations:** 1 Beijing University of Chinese Medicine, Beijing, 100102 China; 2 State Key Laboratory of Generic Manufacture Technology of Chinese Traditional Medicine, Linyi, 276000 China

**Keywords:** Bioinformatics, Biochemical networks

## Abstract

Yinzhihuang granules (YZHG) is a patented Chinese medicine for the treatment of hepatitis B. This study aimed to investigate the intrinsic mechanisms of YZHG in the treatment of hepatitis B and to provide new evidence and insights for its clinical application. The chemical compounds of YZHG were searched in the CNKI and PUBMED databases, and their putative targets were then predicted through a search of the SuperPred and Swiss Target Prediction databases. In addition, the targets of hepatitis B were obtained from TTD, PharmGKB and DisGeNET. The abovementioned data were visualized using Cytoscape 3.7.1, and network construction identified a total of 13 potential targets of YZHG in the treatment of hepatitis B. Molecular docking verification showed that CDK6, CDK2, TP53 and BRCA1 might be strongly correlated with hepatitis B treatment. Furthermore, GO and KEGG analyses indicated that the treatment of hepatitis B by YZHG might be related to positive regulation of transcription, positive regulation of gene expression, the hepatitis B pathway and the viral carcinogenesis pathway. Network pharmacology intuitively shows the multicomponent, multitarget and multichannel pharmacological effects of YZHG in the treatment of hepatitis B and provides a scientific basis for its mechanism of action.

## Introduction

The cause of hepatitis B is hepatitis B virus (HBV), which is a double-stranded, circular, incompletely closed DNA virus^1–3^. Antiviral therapy involving pegylated interferon or nucleoside analogs (lamivudine, adefovir, entecavir, tenofovir disoproxil, or tenofovir alafenamide) is currently provided clinically to patients with hepatitis B to inhibit HBV DNA replication and improve liver inflammation and fibrosis^4,5^, but this treatment protocol requires patients to be treated indefinitely and has a relatively low cure rate. Researchers are constantly searching for more effective drugs. In addition, the long-term use of nucleos(t)ide analogs (NAs) might lead to drug resistance and renal damage and cannot reverse liver fibrosis. Therefore, an increasing number of people have begun to focus on Chinese herbal medicines and seek safer and more cost-effective supplementary drugs for hepatitis B^6–8^.

Chinese herbal medicines have a long history of use in China. In recent years, researchers have continuously found effective pharmaceutical ingredients and targets for the treatment of diseases from Chinese herbal medicines. These results show that Chinese herbal medicines constitute extremely rich resources, and the discovery of new medicine sources from Chinese herbal medicine is becoming a major method of drug development. Yinzhihuang granules (YZHG) can clear away heat and toxic materials, promote diuresis and eliminate jaundice. The prescription is composed of "Yinchenhao Decoction" (Han, Zhongjing Zhang, "Treatise on Febrile Diseases") and "Huanglianjiedu Decoction" (Tang, Tao Wang, "Essential Secrets from Outside the Metropolis"). The YZHG formula contains four herbs, namely, Artemisia scopatia Waldst. et Kit. (or Antemha capillaris Thunb.), Gardenia jasminoides Elli, Scutellaria baicalensis Georgi, and Lonicera japonica Thunb. All of these herbs are widely used in clinical practice for the treatment of hepatitis^9–12^.

**Figure 1:**
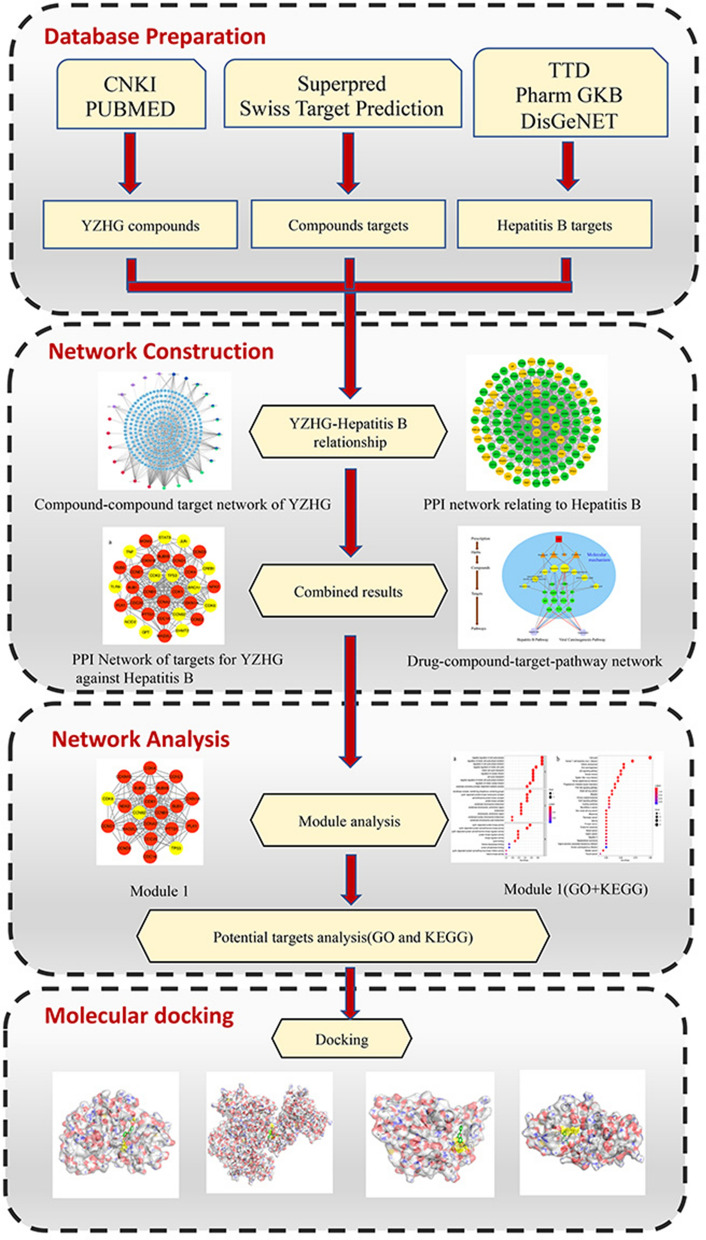
Workflow diagram of the network pharmacology-based analysis of YZHG in the treatment of hepatitis B.

A single Chinese herbal medicine cannot achieve the synergistic effect of all the drugs in the formula. Therefore, a single drug in the YZHG formula might be responsible for the therapeutic effect through different targets and pathways, and different ingredients might also act on the same target. Network pharmacology integrates multidisciplinary technologies and contents, such as multidirectional pharmacology, computational biology, and network analysis. A comprehensive network analysis of drug effects can be realized using multitarget research strategies^13,14^. Traditional Chinese medicines and their prescriptions work synergistically through multiple components, multiple pathways, and multiple targets. Thus, the research strategy of network pharmacology coincides with the application principle of traditional Chinese medicines^15^. Therefore, we simulated the mechanism of action of YZHG on hepatitis B based on a synthetic drug-ingredient-target-pathway network. The relevant workflow is shown in Fig. 1.

## Results

### Compound-putative target network

**Figure 2:**
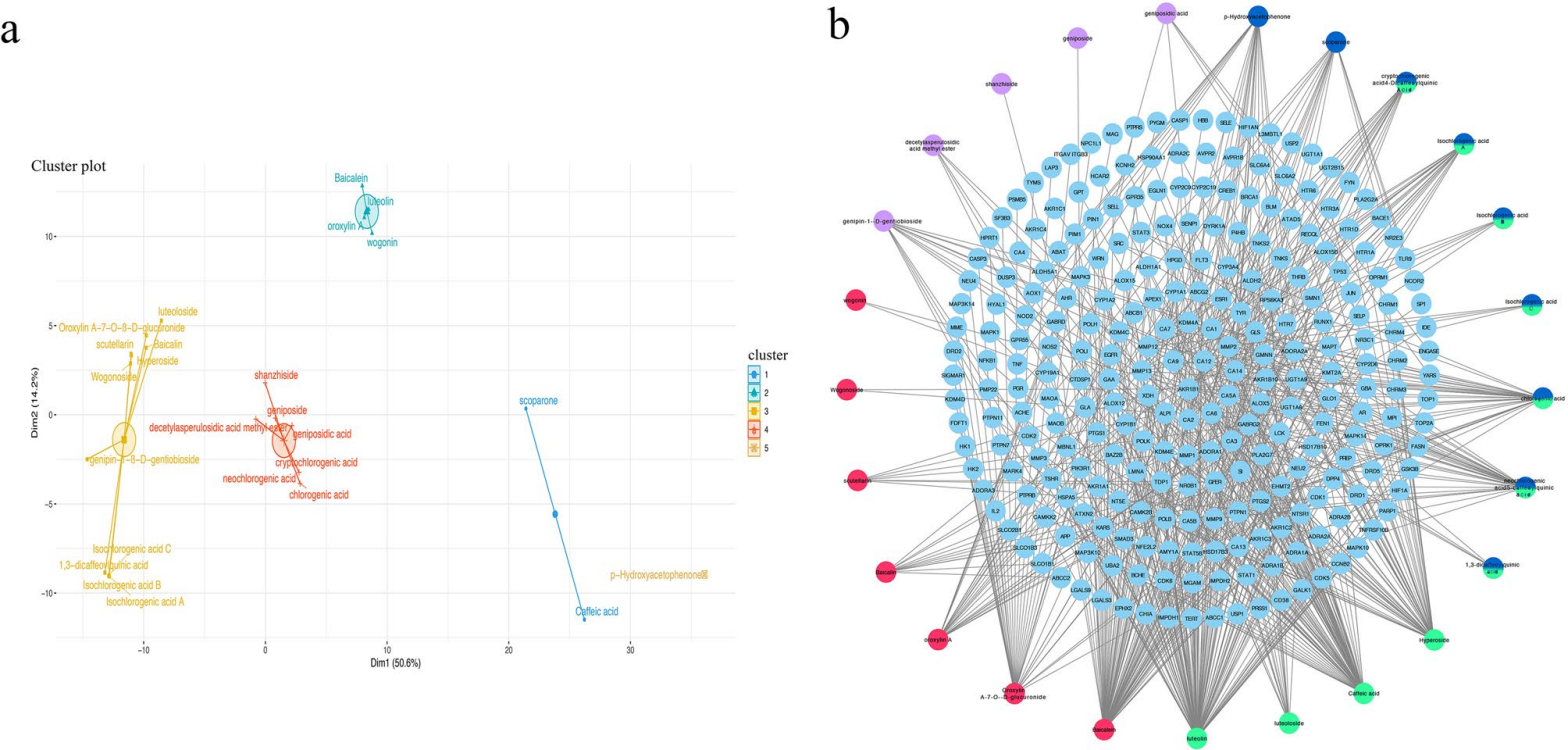
(**a**) Principal component analysis of chemical compounds of YZHG. (**b**) Compound-putative target network of YZHG. The light blue color indicates the chemical composition; the red color indicates the composition derived from Huangqin; the purple color indicates the composition derived from Zhizi; the dark blue color indicates the composition derived from Yinchen; the green indicates the composition derived from Jinyinhua; and the blue-green gradient indicates the compounds derived from Yinchen and Jinyinhua [drawn by Cytoscape 3.7.1 (https://www.cytoscape.org/)].

The details of the 25 compounds in YZHG are described in Supplementary Table S1. A cluster analysis of 25 components derived from four Chinese herbal medicines was performed. The different regions in Fig. 2a show a wide distribution range, which indicates that the 25 components of the four Chinese medicines are very dispersed, and the effects of traditional Chinese medicines are widely distributed. The ingredients in YZHG can play a role in the treatment of diseases through a synergistic action. As shown in Fig. 2b, the compound-putative target network included 281 nodes (25 compound nodes and 256 putative target nodes) and 614 edges. In addition, the network analysis showed that the average degree value of the compounds was 24.56, which indicated that YZHG have multiple targets in the treatment of hepatitis B. Notably, the network contained eight compounds with degrees ≥ 24.56, and the top three compounds, which play significant roles in the network, were luteolin (degree = 120), baicalein (degree = 86), and caffeic acid (degree = 80).

### PPI network of hepatitis B targets

**Figure 3:**
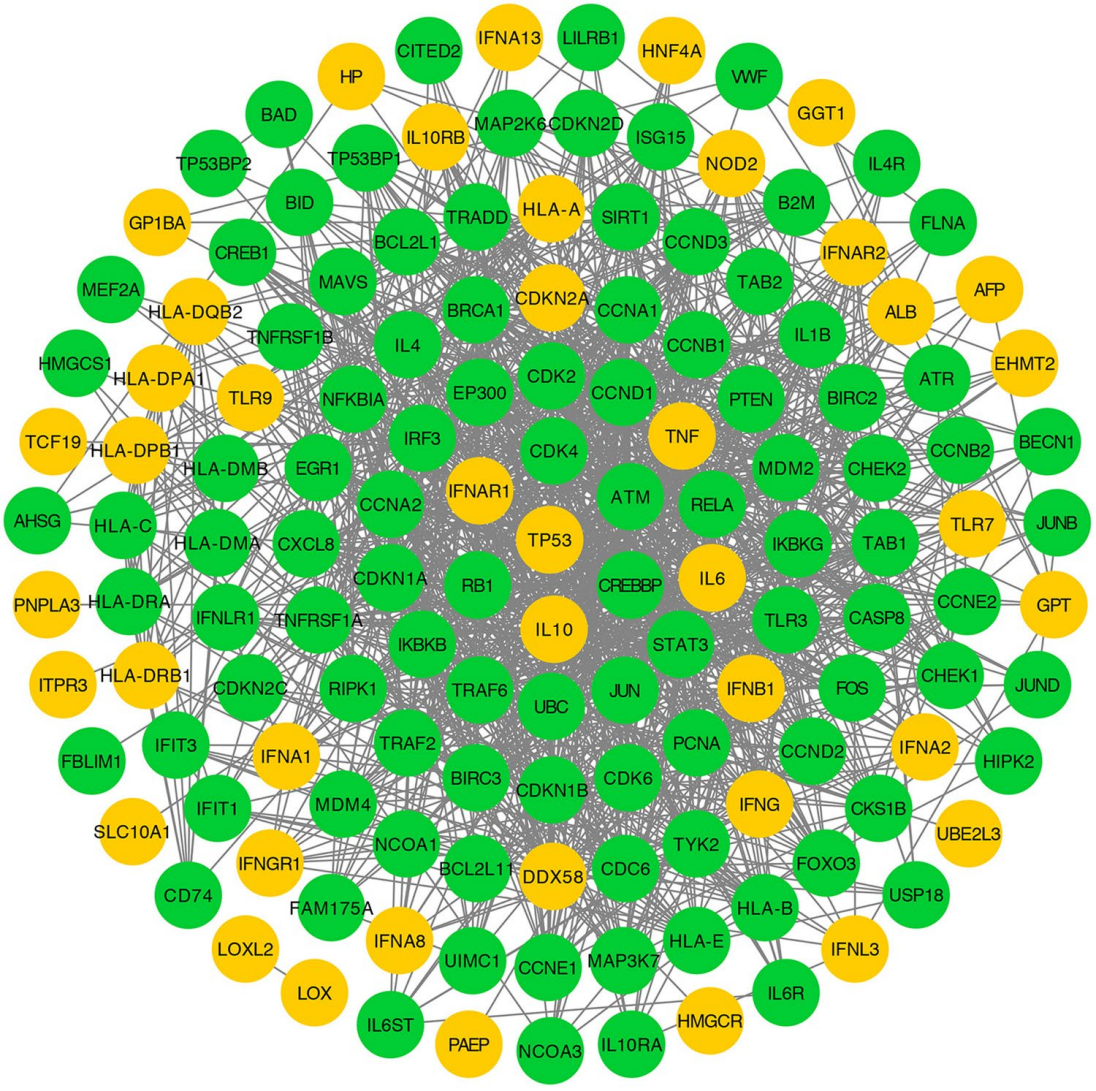
PPI network related to hepatitis B. The orange color indicates primary proteins, and the green color indicates secondary proteins [drawn by Cytoscape 3.7.1 (https://www.cytoscape.org/)].

A total of 42 hepatitis B targets were retrieved from the TTD, PharmGKB and DisGeNET databases (as shown in Supplementary Table S2). The PPI network of these hepatitis B targets was built with 143 nodes (42 hepatitis B targets and 101 other human proteins that interacted with hepatitis B targets) and 1168 edges (Fig. 3). Based on the median values for degree, betweenness centrality, and closeness centrality, which were 16.3356643, 0.01004104, and 0.43150303, respectively, we identified 27 highly connected nodes (degree > 16.336, betweenness centrality > 0.010, and closeness centrality > 0.432) as significant hepatitis B-related targets.

### PPI network and module analysis of potential targets

**Figure 4:**
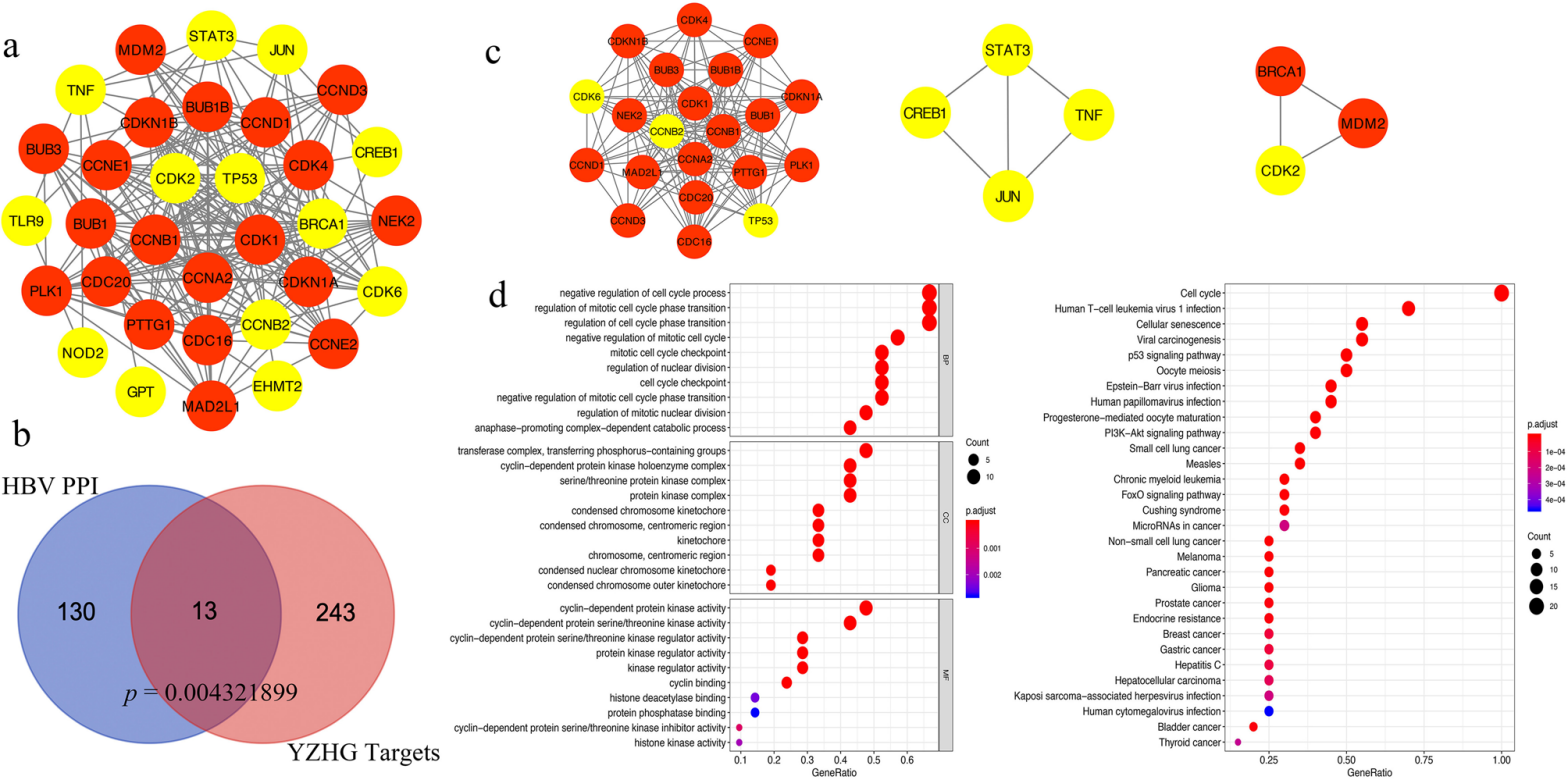
(**a**) PPI network related to potential targets. The yellow color indicates primary proteins, and the red color indicates secondary proteins [drawn by Cytoscape 3.7.1 (https://www.cytoscape.org/)]. (**b)** Venn diagram of protein interactions. (**c**) Module analysis of the network for the identification of potential targets [drawn by Cytoscape 3.7.1 (https://www.cytoscape.org/)]. (**d**) The GO and KEGG diagram of module 1 [drawn by R 3.6.3 (https://cran.r-project.org/doc/FAQ/R-FAQ.html#Citing-R)].

To further unveil the potential pharmacological mechanisms of YZHG against hepatitis B, we intersected the two networks shown in Figs. 2a and 3 to obtain 13 potential targets of YZHG in the treatment of hepatitis B. We then constructed a PPI network of these 13 targets to obtain their corresponding secondary proteins (Fig. 4a). A hypergeometric test of the protein interaction results was performed, which yielded a statistically significant results with a *p* value of 0.004321899 (< 0.05) (Fig. 4b). The MCODE plug-in in Cytoscape 3.7.1 (https://www.cytoscape.org/) was then used for a module analysis of the targets in the PPI network. The clustering module obtained from the module analysis might represent some key characteristics of the PPI network and could have specific biological significance^16^. In this study, the module analysis identified three clustering modules: Module 1, MCODE score = 14.3; Module 2, MCODE score = 3.333; and Module 3, MCODE score = 3 (Fig. 4c). Modules 2 and 3 contained fewer targets, and the analysis did not identify a statistically significant difference. Therefore, Gene Ontology (GO) and Kyoto Encyclopedia of Genes and Genomes (KEGG) analyses were only performed with Module 1, and the results were visualized using the R package (Fig. 4d). The data obtained from the GO analysis of Module 1 indicate that the mechanism of YZHG in the treatment of hepatitis B is mainly related to the following biological processes (BPs), cell components (CCs) and molecular functions (MFs): negative regulation of the cell cycle process and regulation of the mitotic cell cycle phase transition, transferring phosphorous-containing groups and the cyclin-dependent protein kinase holoenzyme complex, and cyclin-dependent protein kinase activity and cyclin-dependent protein serine/threonine kinase activity, respectively. Simultaneously, the KEGG enrichment analysis of Module 1 suggests that the mechanism of YZHG in the treatment of hepatitis B is mainly related to the cell cycle, human T-cell leukemia virus 1 infection, cellular senescence, viral carcinogenesis, and p53 signaling pathway.

### GO and KEGG enrichment analysis

**Figure 5:**
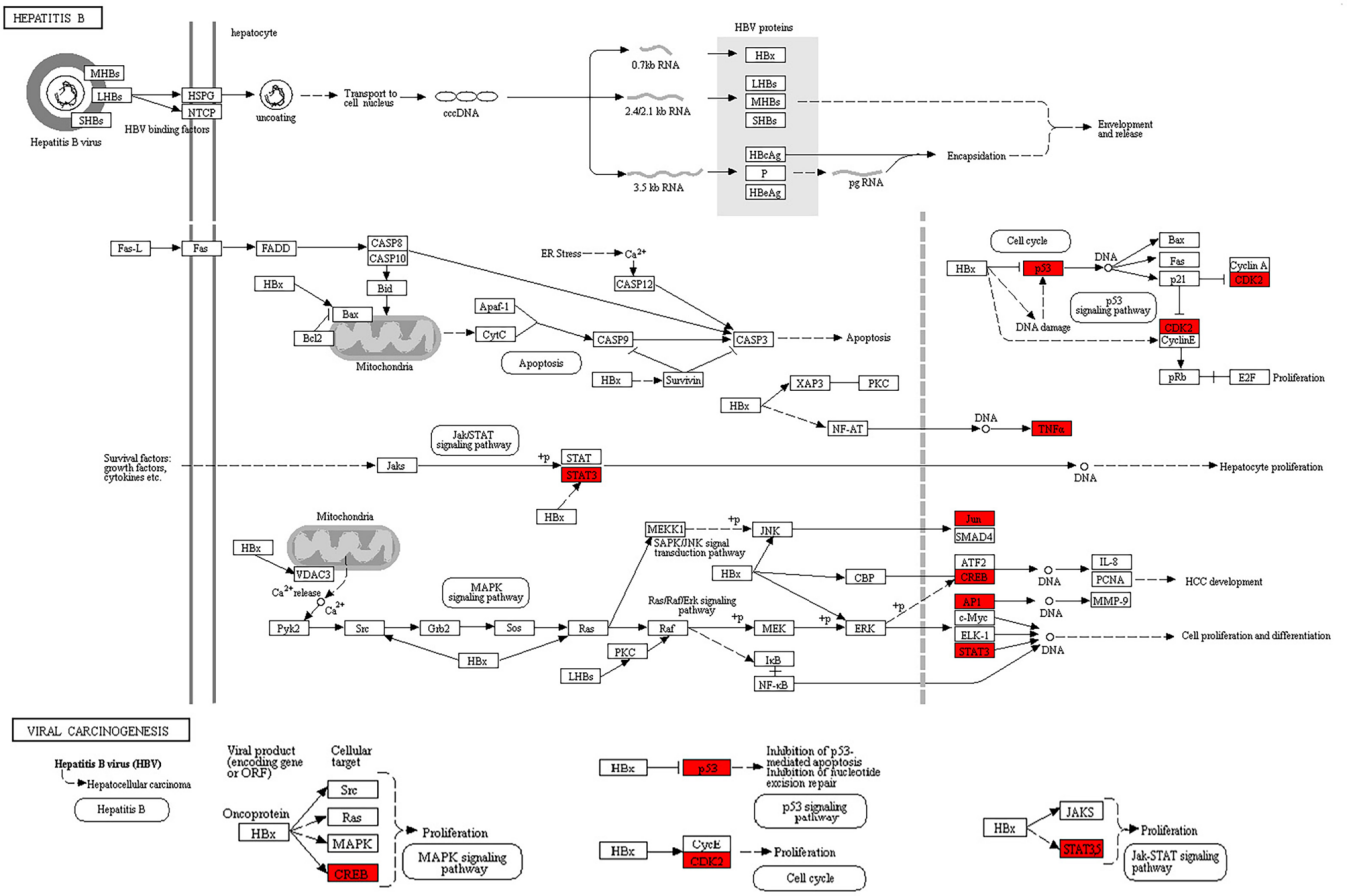
KEGG analysis pathway map of potential targets.

We further analyzed the 13 potential targets of YZHG in the treatment of hepatitis B using the David database. After data screening, four GO enrichment entries (Supplementary Table S3) and two KEGG pathways (Supplementary Table S4) were obtained. The four GO items were positive regulation of transcription, DNA-templated (GO:0045893), positive regulation of gene expression (GO:0010628), positive regulation of transcription from the RNA polymerase II promoter (GO:0045944) and transcription regulatory region DNA binding (GO:0044212). In addition, the two KEGG pathways are hepatitis B (hsa05161) and viral carcinogenesis (hsa05203). Based on the abovementioned data, we further visually analyzed the two KEGG pathways using the Pathview website (Fig. 5). The visual pathway map shows that the potential components of YZHG in the hepatitis B pathway are mainly related to the nuclear membrane of liver cells, and hepatitis B can thus be treated by regulating cell proliferation and differentiation and the progression of hepatocellular carcinoma. With regard to viral carcinogenesis, the potential components of YZHGs in the treatment of hepatitis B mainly regulate cell proliferation through direct or indirect action, which yields a therapeutic effect.

### Molecular docking verification

**Figure 6:**
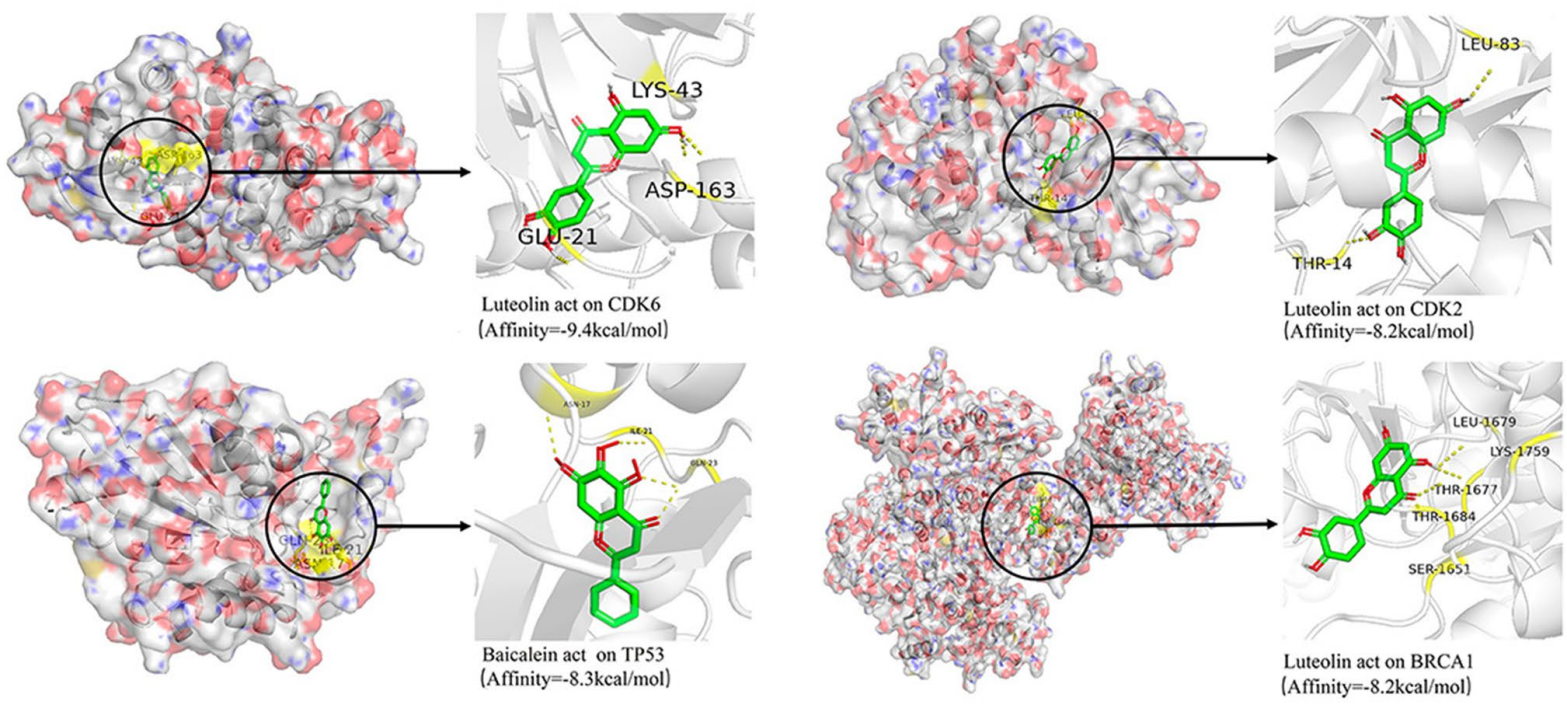
Detailed target-compound interactions with the three highest molecular docking affinities.

The PDB database is powered by the Protein Data Bank and contains archived information on the 3D shapes of proteins, nucleic acids, and complex assemblies that helps researchers understand all aspects of biomedicine, ranging from protein synthesis to health and disease. Therefore, we searched the PDB database for the 3D structures of the 13 potential targets of YZHG in the treatment of hepatitis B and found 3D structures for 10 of the targets (CDK2, GPT, EHMT2, STAT3, JUN, BRCA1, TP53, TNF, CREB, and CDK6); the 3D structures of NOD2 and CCNB2 were not available, and the 3D structure of TLR9 did not have a human source. The abovementioned 10 targets and their corresponding small-molecule drug ligands are docked using AutoDock Vina, an open source program for molecular docking that yields an improved scoring function. The effective optimization and multithreading function of this program improve the speed and accuracy of the docking and thereby markedly improve the average accuracy of the combined mode prediction^17,18^. A grid box size of 40 × 40 × 40 points with a spacing of 1.0 Å between grid points was generated to cover almost the entire favorable protein-binding site. The X, Y and Z centers were adjusted according to different macromolecular forms. As shown in Table 1, a total of 18 pairs of docking results were obtained. The pair with the highest binding affinity (9.4 kcal/mol) was luteolin and CDK6, whereas the pairs with the second- and third-highest binding affinities (8.3 and 8.2 kcal/mol) were baicalein and TP53 and Luteolin and BRCA1/CDK2 docking, respectively. In addition, the positive control drug entecavir, which exerts a therapeutic effect on hepatitis B. And then, obtain its corresponding 2D structure. The targets of entecavir and potential targets of YZHG involved in the treatment of hepatitis B were docked, and the obtained affinity data were used as the baseline data of the positive control. The results showed that of the 18 groups of molecular docking data, only one group (entecavir positive control data) yielded higher values than the YZHG group^3^. This result suggests that their combination might play an important role in the treatment of hepatitis B with YZHG. Detailed information on the interaction of the target compounds obtained with the docking simulations is shown in Fig. 6.

**Table 1:** Docking scores of the active ingredients of YZHG with their potential targets.

Targets	PDB ID	Compounds	Affinity (kcal/mol)	Baseline affinity (kcal/mol)
CDK2	6Q3B	Luteolin	8.2	7.2
GPT	6BW6	Caffeic acid	7.5	6.9
EHMT2	6MM1	Neochlorogenic acid	8.0	6.4
EHMT2	6MM1	Chlorogenic acid	7.8	6.4
EHMT2	6MM1	Caffeic acid	5.9	6.4
EHMT2	6MM1	Luteolin	7.8	6.4
STAT3	6QHD	Neochlorogenic acid	7.3	7
STAT3	6QHD	Chlorogenic acid	7.2	7
JUN	1JNM	Baicalein	5.6	4.8
JUN	1JNM	Oroxylin A	5.5	4.8
BRCA1	4Y18	Baicalein	8.0	6.5
BRCA1	4Y18	Luteolin	8.2	6.5
TP53	3DCY	Baicalein	8.3	7.1
TP53	3DCY	Luteolin	8.0	7.1
TNF	1TNF	p-Hydroxyacetophenone	6.2	8.4
CREB1	2LXT	Baicalein	5.8	4.6
CREB1	2LXT	Oroxylin A	5.7	4.6
CDK6	4AUA	Luteolin	9.4	5.9

### Herb-compound-target-pathway network

**Figure 7:**
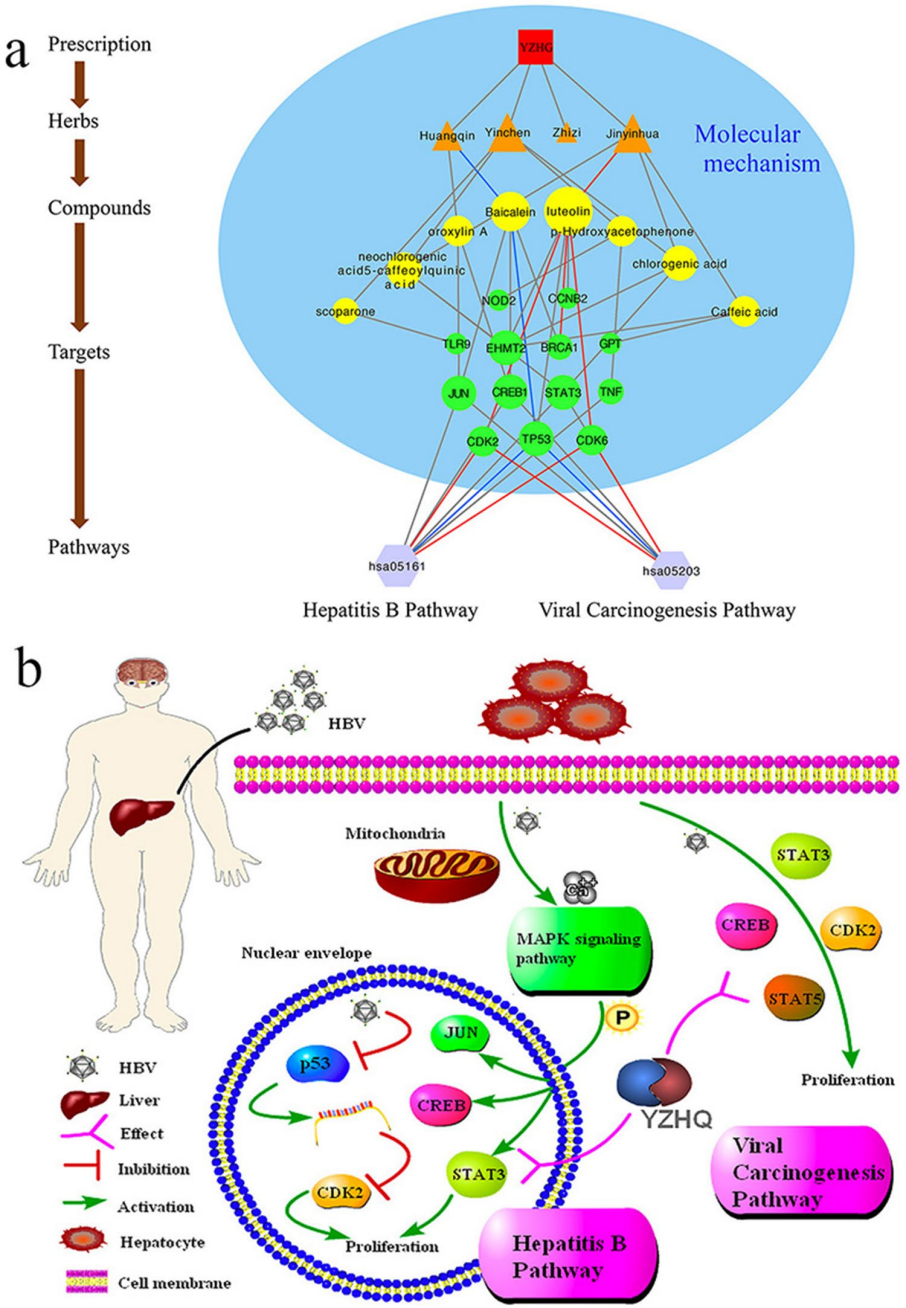
(**a**) Herb-compound-target-pathway network. The red squares represent the YZHG; the orange triangles represent the four drugs in the YZHG; the yellow circles represent the ingredients in each drug; the green circles represent the targets corresponding to the ingredients; and the purple hexagons represent the pathways enriched with the targets. (**b**) Illustration of the crucial biological processes involving putative targets and known therapeutic targets of YZHG: signal transducer and activator of transcription 3, cyclin-dependent kinase 2, transcription factor AP1, cAMP-response element-binding protein, signal transducer and activator of transcription 5, and cellular tumor antigen p53.

Based on the abovementioned information, we then constructed an herb-compound-target-pathway network (Fig. 7a) to holistically explain the mechanism of YZHG in the treatment of hepatitis B. In the figure, the red squares represent the YZHG, the orange triangles represent the four drugs in the YZHG, the yellow circles represent the ingredients in each drug, the green circles represent the targets of the ingredients, and the purple hexagons represent the pathways enriched in the targets. Based on the molecular docking results, we highlight the positions of CDK6, CDK2, BRCA1 and TP53 in the figure. The size of the graphic is proportional to the degree value, and the important targets are enriched in the hepatitis B pathway and viral carcinogenesis pathway. Adopting a system pharmacology-based approach, we uncovered the working mechanism of YZHG in the treatment of hepatitis B by exploring key active compounds, targets, and pathways (Fig. 7b).

## Discussion

Approximately 259 million people worldwide are infected with HBV, which causes hepatitis B, one of the top ten causes of death worldwide. Specifically, hepatitis B causes 890,000 deaths every year and can thus be considered a serious problem that affects people’s lives and health. Drugs have been clinically used for the treatment of hepatitis B. Interferon and nucleoside analogs can inhibit viral replication but cannot cure the disease. Researchers have developed vaccines that can effectively prevent hepatitis B, but these vaccines do not help patients who are already infected with the virus^19^. Therefore, patients need to use these drugs for a long time, which leads to drug resistance and adverse side effects^20,21^. Researchers have agreed on the current strategy for the treatment of hepatitis B and suggest that the use of loopholes in the replication cycle to treat infections and enhance the adaptive immune response to the innate immune response can result in the effective treatment of patients with hepatitis B^22–24^. Based on the complex mechanism of action of hepatitis B, single-target drugs cannot easily yield better therapeutic effects, and multitarget combination drug therapy has become the trend in the development of future treatments for hepatitis B^25^. Therefore, in this study, we explored the mechanism of the patented Chinese medicine YZHG as an adjuvant treatment for hepatitis B through network pharmacology to provide a reference for further research.

According to the screening degree in the compound-putative target network, the top three compounds, namely, luteolin, baicalein and caffeic acid, are the important compounds in YZHG with stronger correlations. Luteolin was identified as the component with the highest degree, and our further research suggests that this component is related to several hepatitis B targets, including CDK2, CDK6, EHMT2, STAT3, BRCA1, TP53 and CCNB2. A recent study involving in vitro experiments confirmed that luteolin can establish a signal cascade composed of ERK and HNF4α for the treatment of hepatitis B, and these findings have been verified through in vivo experiments, which shows that luteolin can inhibit HBV replication and can be used to fight hepatitis B^26^. In addition, other studies have shown that the luteolin derivative luteolin-6-C-β-D-boivinopyranosyl-3′-O-β-D-glucopyranoside can show significant anti-HBV activities by specifically inhibiting the secretion of HBsAg in HepG2.2.15 cells^27^. Previous studies have also shown that the DNA level of anti-entecavir (ETV)-resistant HBV is lower compared with that obtained with baicalein^28^. In addition, the activity of HBV in HepG2 2.2.15 cells has been studied using a new baicalein derivative, and compounds 4k and 4h were found to be effective anti-HBV agents^29^. Caffeic acid is also the main chemical substance used for the treatment of hepatitis B. Some researchers have used lamivudine as a positive control to study the effect of caffeic acid on the secretion of HBsAg and HBeAg, and the results have shown that caffeic acid exerts a strong inhibitory effect^30^.

Combining the compound-putative target network with the PPI network of hepatitis B targets, a total of 13 potential targets were obtained, namely, CDK2, TLR9, GPT, NOD2, EHMT2, STAT3, JUN, BRCA1, TP53, CCNB2, TNF, CREB1 and CDK6. The PPI network was constructed from 13 targets, and a module analysis identified three modules. Because the numbers of targets in Modules 2 and 3 were very small, their analysis would not be instructive; thus, we perform GO and KEGG analyses only using Module 1. The primary proteins in Module 1 are CDK6, CCNB2, and TP53. Interestingly, the molecular docking of CDK6 and luteolin and TP53 and baicalein exhibited the highest affinities, which indicates that CDK6 and TP53 might be highly correlated in the treatment of hepatitis B with YZHG.

Cyclin-dependent kinases (CDKs) are a set of Ser/Thr kinase systems that correspond to the cell cycle process. Various CDKs are alternately activated along the phase of the cell cycle by phosphorylating the corresponding substrates, which allows the orderly progression of cell cycle events^31,32^. CDK2 regulates the S phase of the cell cycle, and CDK4 and CDK6 regulate the Gap 1 (G1) and synthesis (S) phases of the cell cycle^33^. The cyclin D2/CDK4 and cyclin D2/CDK6 complexes play key roles in controlling the cell cycle from the G1 to the S phase. Moreover, some researchers have shown that Cdk4-cyclin D and Cdk6-cyclin D phosphorylate the tumor suppressor retinoblastoma (Rb) protein in mammalian cells. The phosphorylation of Rb leads to the release of E2F, which results in the transition from the G1 phase to the S phase required for gene transcription^34^. This finding indicates that inhibition of the phosphorylation of the abovementioned targets might inhibit the cell cycle progression of liver cells and the replication of hepatitis B virus. CDK6 regulates the transcription of many genes, and its function might depend on its kinase activity^35,36^. Its transcriptional function is very important for the maintenance of hematopoietic stem cells and leukemic stem cells and its role in promoting myeloid and lymphoid malignancies (including AML)^37–41^. The potential application of CDK4/6 inhibitors has been widely recognized, and CDK4/6 inhibitors are considered a major breakthrough in cancer treatment^42–44^. The analysis of Module 1 revealed that CDK1 and CDK4, in addition to CDK6, also belong to the CDK family, which indicates that they might be responsible for the synergistic effects of YZHG on the treatment of hepatitis B. For example, the study suggests that CDK4 is involved not only in the process from a normal liver to chronic hepatitis but also during the transition to HCC^45^. The above-described evidence indicates that the effect of YZHG in the treatment of hepatitis B might be caused by inhibition of the CDK family.

TP53 is also called the cell tumor antigen p53. HBV is the major risk factor for hepatocellular carcinoma (HCC), and blocking the progression of hepatitis B to HCC might inhibit the occurrence of HCC. The results of a previous study indicate the importance of TP53 in the pathogenesis of HCC^46^. Notably, researchers have detected a high frequency of the hot spot R249S mutation of TP53 in tumor tissues. The R249S mutation is induced by aflatoxin metabolites, and this TP53 mutation can interact with HBx to induce cell proliferation^47–49^. Studies have shown that TP53 might play an important role in the development of hepatitis into HCC. In addition, studies of HBV-induced infections and the microRNA profiles in HCC have revealed that TP53, a gene that exerts a major impact on HCC and HBV infection, is the most frequently altered gene in HBV-associated HCC. On the one hand, TP53 is only associated with shorter survival in HBV-associated HCC, and on the other hand, TP53 is a classical suppressor gene involved in the accumulation of the cell cycle and genetic changes^50^.

The targets included in Module 1 were subjected to GO and KEGG enrichment analyses using the R package. In the GO analysis, we filtered the top 10 entries in the BP, CC, and MF categories based on the following criteria: *p* value < 0.01 and q-value < 0.05. The top-ranked entry in the BP category is negative regulation of the cell cycle process, the top-ranked entry in the CC category is transferring phosphorus-containing groups, and the top-ranked entries in the MF category are cyclin-dependent protein kinase activity and cyclin-dependent protein serine/threonine kinase regulator activity. Furthermore, we screened the top 30 entries obtained from the KEGG enrichment analysis based on the following criteria: *p* value < 0.05 and *q*-value < 0.05. The top five entries were found to be cell cycle, human T-cell leukemia virus 1 infection, cellular senescence, viral carcinogenesis and p53 signaling pathway. The KEGG enrichment analysis of the 13 potential targets identified two pathways: hepatitis B (hsa05161) and viral carcinogenesis (hsa05203). TNF, JUN, CREB1, TP53, CDK6, STAT3, CDK2, and seven targets are enriched in the hepatitis B pathway. As shown in the KEGG diagram, the potential targets mainly act in the nucleus, where they regulate the cell cycle, cell proliferation and differentiation, and HCC development. For example, as shown in the diagram of the cell cycle, HBx regulates the activity of p53 by indirectly damaging DNA or directly inhibiting the nucleus and thereby transcriptionally activating p21 and inhibiting the expression of the target gene CDK2. In addition, previous studies have suggested that HBV continues to replicate in resting hepatocytes, and this process does not slow down until the hepatocytes begin to divide^51,52^. Researchers have found that HBV induces in HepG2.2.15 cell cycle arrest by regulating the expression of genes related to the G1/S transition^53^. On the one hand, YZHG might play a therapeutic role by regulating p53 and CDK2 in the cell cycle, promoting the cell cycle and inhibiting HBV replication, and on the other hand, a total of six targets were enriched in the viral carcinogenesis pathway. YZHG control the transition from hepatitis B to HCC by affecting the proliferation of hepatocytes through the regulation of CREB, TP53, CDK2, and STAT3.

## Methods

### Data preparation

#### Chemical compounds of YZHG

A search of the literature on YZHG in CNKI and PUBMED^54–57^ identified 25 compounds of YZHG for further study. We entered all of these compounds into the PubChem database^58^ (https://pubchem.ncbi.nlm.nih.gov) and obtained their canonical simplified molecular input entry specification (SMILES) information. The compounds with no canonical SMILES information were drawn using ChemDraw^59^. Structural information of all 25 compounds was collected, and a cluster analysis was performed based on the molecular descriptors of the compounds. K-means clustering of the chemical components was performed using the factoextra software package^60^.

#### Putative targets of YZHG

The SMILES information of the 25 identified compounds was imported into the Search Tool SuperPred and SwissTargetPrediction databases. The SuperPred web server^61, 62^ (https://prediction.charite.de/) connects the chemical similarity of drug-like compounds with molecular targets and the therapeutic approach based on the similar property principle. For query compounds with sufficient structural similarity, the web server provides predictions of the medical indication areas of novel compounds and the identification of new leads for known targets. SwissTargetPrediction^63^ (https://www.swisstargetprediction.ch/) is a web tool (online since 2014) that aims to predict the most likely protein targets of small molecules, and the predictions are based on the similarity principle through reverse screening.

#### Hepatitis B targets

Different genes related to hepatitis B were gathered from three resources. (1) The Therapeutic Target Database^64^ (TTD, https://db.idrblab.org/ttd/) is a database that provides known information for exploring therapeutic protein and nucleic acid targets, the targeted disease, pathway information, and the corresponding drugs directed at each of these targets. We screened the TTD using the keyword “Hepatitis B” and obtained 18 known hepatitis B-related targets. (2) The Pharmacogenomics Knowledgebase^65^ (PharmGKB, https://www.pharmgkb.org/) is a resource that collects, curates, and disseminates information on the impact of human genetic variations on drug responses. We searched PharmGKB using the keyword “Hepatitis B” and acquired one known Hepatitis B-related target. (3) DisGeNET^66^ (https://www.disgenet.org/) is a comprehensive discovery platform developed for addressing diverse questions concerning the genetic underpinning of human diseases. We searched the platform using the keyword “Hepatitis B” and selected 76 genes with a gene-disease score ≥ 0.1. After redundant information and microRNAs were deleted, 42 known hepatitis B-related targets were collected.

#### Protein–protein interaction data

Protein–protein interaction (PPI) data were extracted from the Search Tool for the Retrieval of Interacting Genes/Proteins (STRING, https://string-db.org/). The STRING database aims to collect, score, and integrate all publicly available sources of protein–protein interaction information, and to complement these with computational predictions. Its goal is to achieve a comprehensive and objective global network, including direct (physical) as well as indirect (functional) interactions^67^. STRING defines PPIs with confidence ranges for data scores (low: < 0.4; medium: 0.4 to 0.7; high: > 0.7). We inputted the hepatitis B-related targets into the STRING database, with the species limited to “Homo sapiens” and confidence scores higher than 0.7. The hypergeometric test was conducted using the phyper function in R 3.6.3 (https://cran.r-project.org/doc/FAQ/R-FAQ.html#Citing-R).

### Network construction

In this study, we constructed five networks. First, (1) a compound-putative target network was established by linking the chemical compounds of YZHG with their corresponding targets; (2) the PPI network of hepatitis B targets was built by connecting hepatitis B-related targets and other human proteins that are linked to or interacted with hepatitis B targets; and (3) a compound-hepatitis B target network was constructed by intersecting the compound-putative target network and the PPI network of hepatitis B targets. The genes that did not intersect were removed; specifically, the potential targets between the compound-putative target network and the PPI network of hepatitis B targets were the potential targets of the ingredients of YZHG in hepatitis B. In addition, (4) a potential target-PPI network was constructed, and a module analysis of the network was performed. Subsequently, (5) an herb-compound-target-pathway network was built by linking herbs, compounds, corresponding targets, and pathways. All of the above networks were constructed using Cytoscape 3.7.1 (https://www.cytoscape.org/)^68–70^, which is a software package for visualizing and analyzing networks. To represent large biological datasets in an easily interpretable manner, biological information is frequently visualized as graphs, i.e., a set of nodes and edges. Nodes can represent biological molecules, and edges connect the nodes depicting some type of relationship. MCODE is a plugin that yields a network based on vertex weights calculated using the local neighborhood density and outward traversal from a locally dense seed protein for isolation of the dense regions^71^.

### GO and KEGG pathway analyses

To illustrate the role of the potential targets in gene function and signal pathways, we used the Database for Annotation, Visualization and Integrated Discovery^72, 73^ (DAVID, https://david.ncifcrf.gov/) for GO function enrichment analysis and KEGG pathway enrichment analysis of the genes in the compound-hepatitis B target network. In addition, a visual KEGG image can be obtained using Pathview^74, 75^ (https://pathview.uncc.edu/home). The GO database (https://geneontology.org/), which includes BP, CC, and MF terms, was used to identify the possible biological mechanisms based on high-throughput genome or transcriptome data^76^. The KEGG (Kyoto Encyclopedia of Genes and Genomes; https://www.kegg.jp/ or https://www.genome.jp/kegg/) is a reference knowledgebase for the biological interpretation of genome sequences and other high-throughput data^77, 78^. Furthermore, the potential target modules of GO and KEGG pathway analysis were performed using Bioconductor clusterProfiler, org.Hs.eg.db and DOSE, which are three R packages used for the enrichment analysis of gene clusters^79^.

### Molecular docking simulation

The binding of the potential target and its corresponding components was evaluated by molecular docking^80^. Molecular docking simulations of potential targets and their corresponding components were performed using AutoDock 4.2 and AutoDock Vina software (Scripps Research Institute) according to published methods^81^. The macromolecular protein target receptors were obtained from the RCSB PDB database (https://www.rcsb.org), and the 2D structures of the small-molecule compound components were obtained from the PubChem Database (https://pubchem.ncbi.nlm.nih.gov).

## Supplementary information

Supplementary file1

## Data Availability

The relevant drug targets, disease targets and pathway names are available in the Supplementary Source files.
