# Supplementary material for: A bioinformatics investigation into molecular mechanism of Yinzhihuang granules for treating hepatitis B by network pharmacology and molecular docking verification
Source: Sci Rep. 2020 Jul 10;10:11448. doi: 10.1038/s41598-020-68224-7 (PMC7351787; doi:10.1038/s41598-020-68224-7)
Supplement: Supplementary file 1 — Supplementary file1 [file 41598_2020_68224_MOESM1_ESM.docx]

**A bioinformatics investigation into molecular mechanism of Yinzhihuang Granules in the treatment of Hepatitis B by network pharmacology and molecular docking approaches**

**Jingyuan Zhang**1,+**, Xinkui Liu**1,+**, Wei Zhou**1**, Guoliang Cheng**2**, Jiarui Wu**1,***, Siyu Guo**1**, Shanshan Jia**1**, Yingying Liu**1**, Bingbing Li**1**, Xiaomeng Zhang**1 **and Miaomiao Wang**1

1Beijing University of Chinese Medicine, Beijing, 100102, China

2 State Key Laboratory of Generic Manufacture Technology of Chinese Traditional Medicine, Linyi, 276000, China

* Corresponding email: exogamy@163.com

+these authors contributed equally to this work

Supplementary Table

| Compound | Structure | Herb | Source | Article |
| --- | --- | --- | --- | --- |
| shanzhiside | 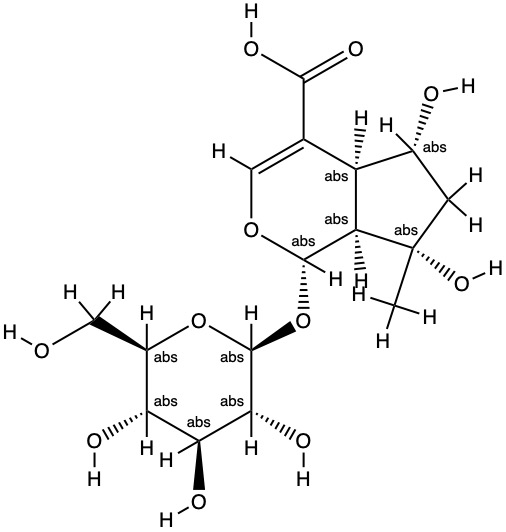 | Zhizi | CNKI | Study on HPLC fingerprints of Yinzhihuang granules and attributive analysis of their common peaks |
| decetylasperulosidic acid methyl ester | 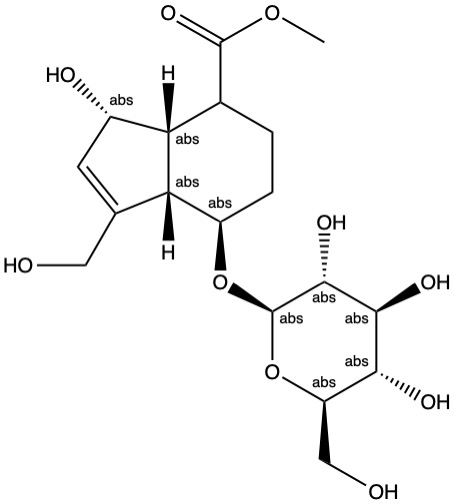 | Zhizi | CNKI | Study on HPLC fingerprints of Yinzhihuang granules and attributive analysis of their common peaks |
| neochlorogenic acid（5-caffeoylquinic acid） | 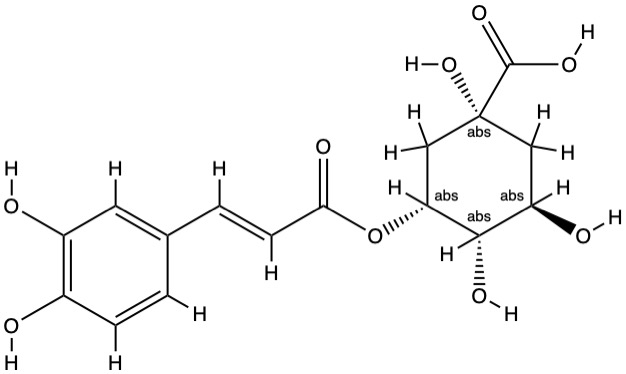 | Yinchen/Jinyinhua | CNKI | Study on HPLC fingerprints of Yinzhihuang granules and attributive analysis of their common peaks |
| chlorogenic acid | 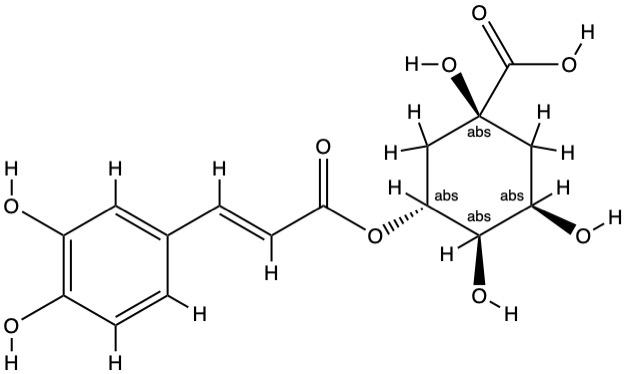 | Yinchen/Jinyinhua | CNKI | Study on HPLC fingerprints of Yinzhihuang granules and attributive analysis of their common peaks |
| cryptochlorogenic acid（4-Dicaffeoylquinic Acid） | 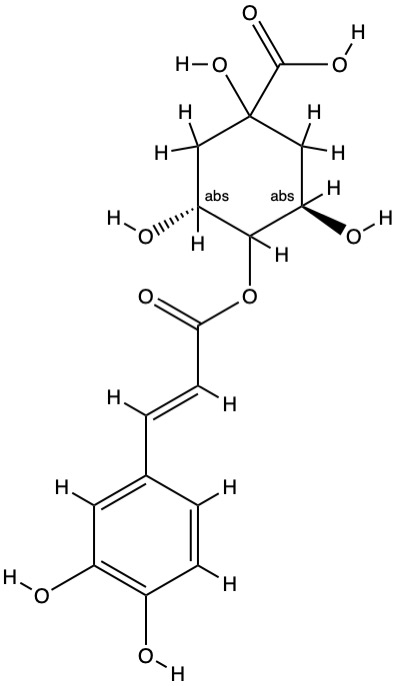 | Yinchen/Jinyinhua | CNKI | Study on HPLC fingerprints of Yinzhihuang granules and attributive analysis of their common peaks |
| genipin-1-β-D-gentiobioside | 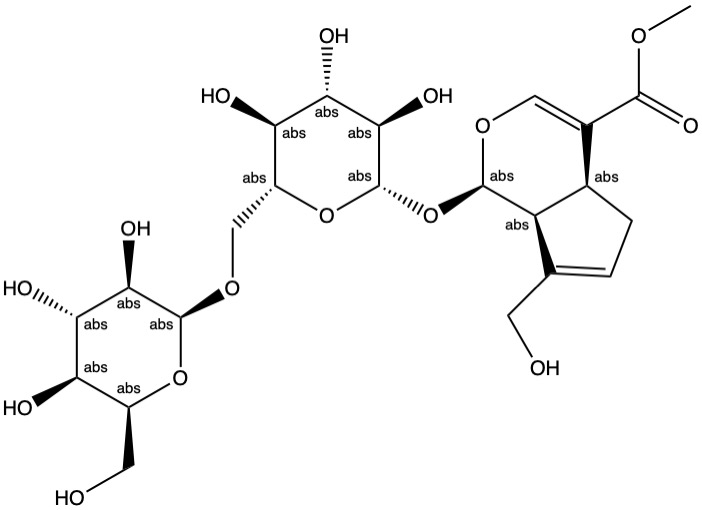 | Zhizi | CNKI | Study on HPLC fingerprints of Yinzhihuang granules and attributive analysis of their common peaks |
| geniposide | 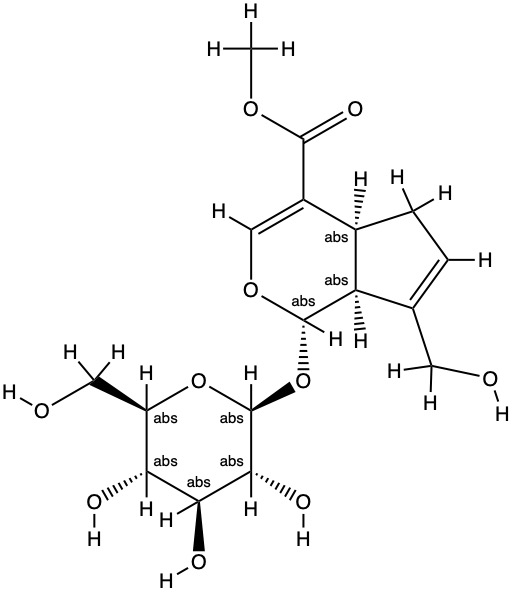 | Zhizi | CNKI | Study on HPLC fingerprints of Yinzhihuang granules and attributive analysis of their common peaks |
| p-Hydroxyacetophenone | 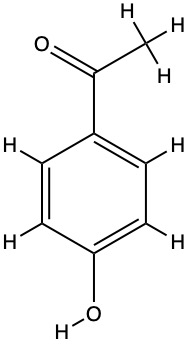 | Yinchen | CNKI | Study on HPLC fingerprints of Yinzhihuang granules and attributive analysis of their common peaks |
| scutellarin | 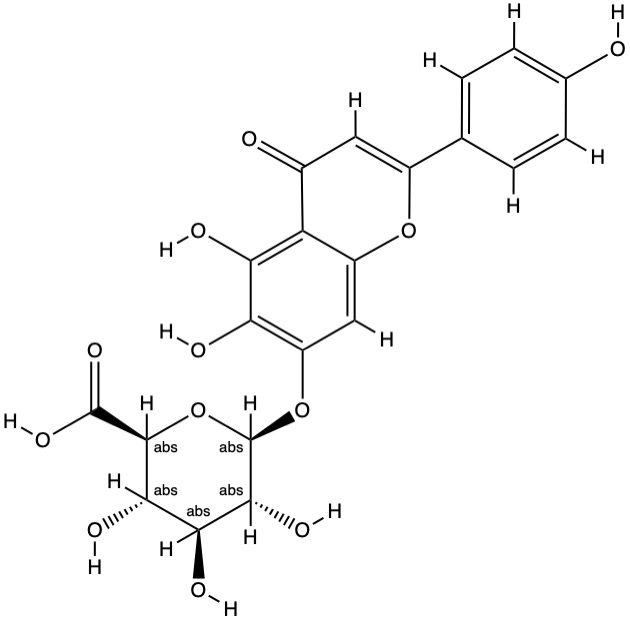 | Huangqin | CNKI | Study on HPLC fingerprints of Yinzhihuang granules and attributive analysis of their common peaks |
| Isochlorogenic acid B | 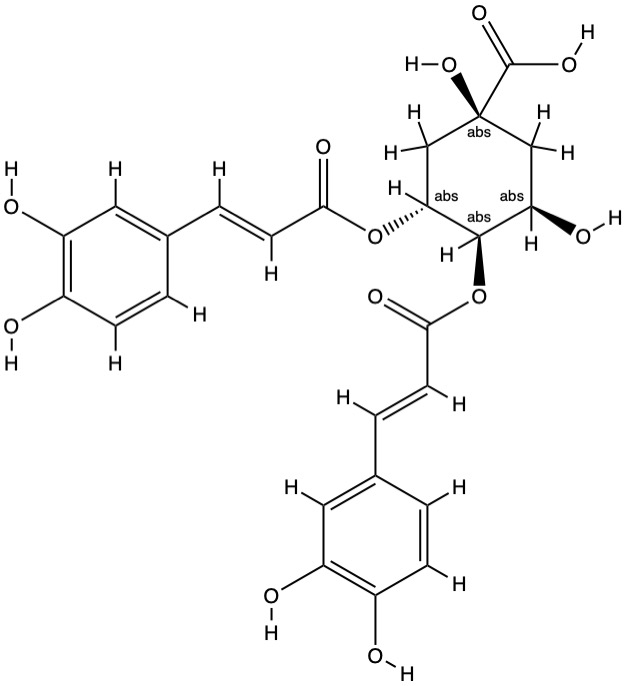 | Yinchen/Jinyinhua | CNKI | Study on HPLC fingerprints of Yinzhihuang granules and attributive analysis of their common peaks |
| Isochlorogenic acid A | 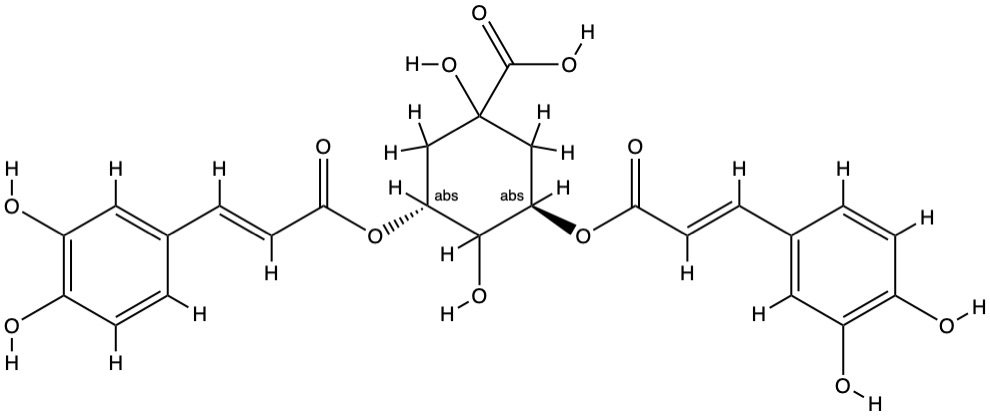 | Yinchen/Jinyinhua | CNKI | Study on HPLC fingerprints of Yinzhihuang granules and attributive analysis of their common peaks |
| Isochlorogenic acid C | 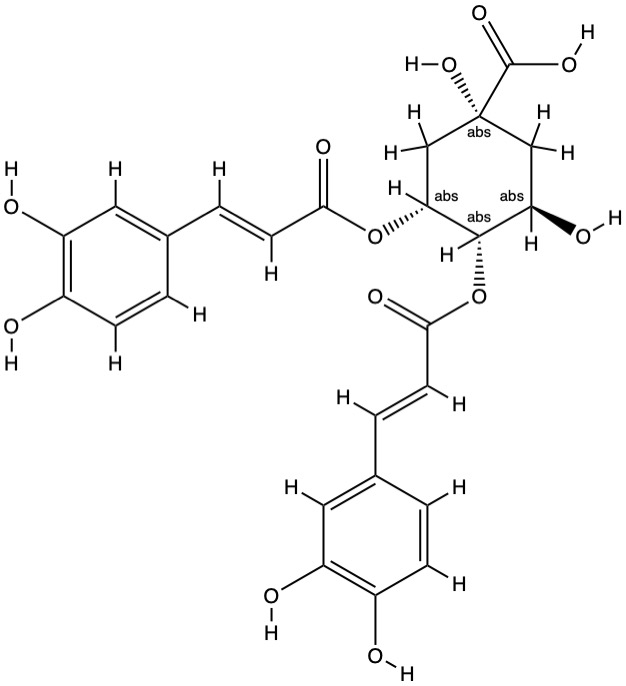 | Yinchen/Jinyinhua | CNKI | Study on HPLC fingerprints of Yinzhihuang granules and attributive analysis of their common peaks |
| Baicalin | 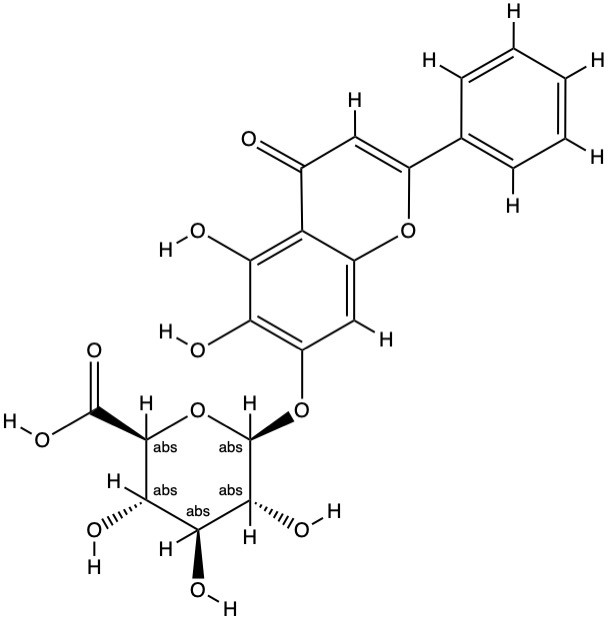 | Huangqin | CNKI | Study on HPLC fingerprints of Yinzhihuang granules and attributive analysis of their common peaks |
| Oroxylin A-7-O-β-D-glucuronide | 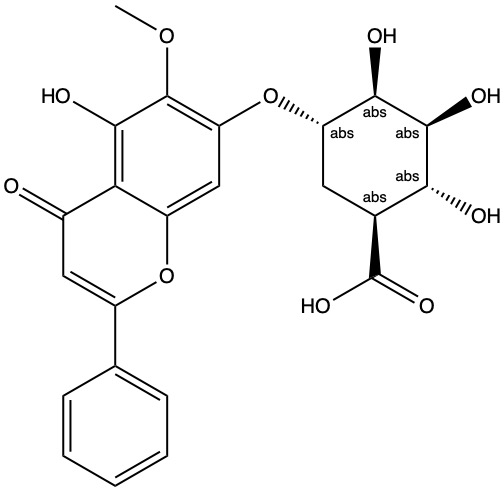 | Huangqin | CNKI | Study on HPLC fingerprints of Yinzhihuang granules and attributive analysis of their common peaks |
| Wogonoside | 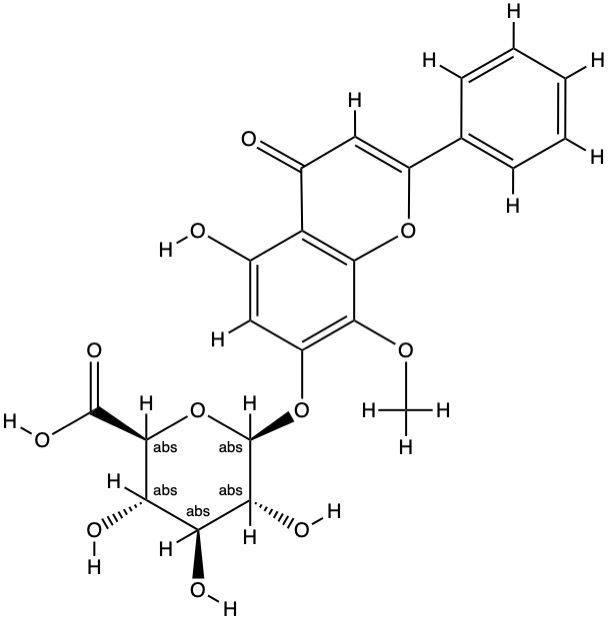 | Huangqin | CNKI | Study on HPLC fingerprints of Yinzhihuang granules and attributive analysis of their common peaks |
| Baicalein | 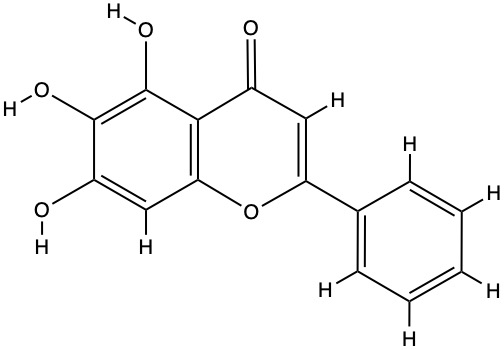 | Huangqin | CNKI | Study on HPLC fingerprints of Yinzhihuang granules and attributive analysis of their common peaks |
| 1,3-dicaffeoylquinic acid | 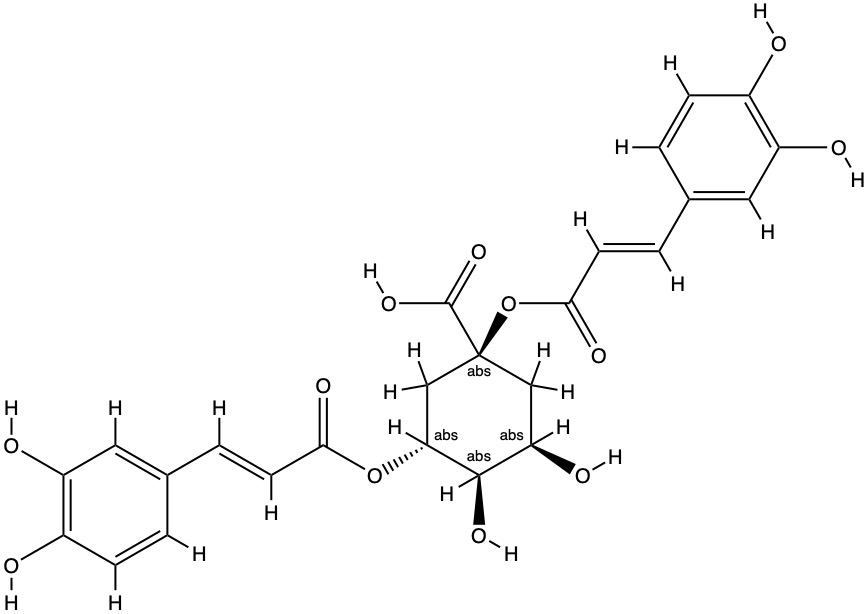 | Yinchen/Jinyinhua | CNKI | Simultaneous determination of fourteen constituents in Yinzhihuang granules by HPLC |
| scoparone | 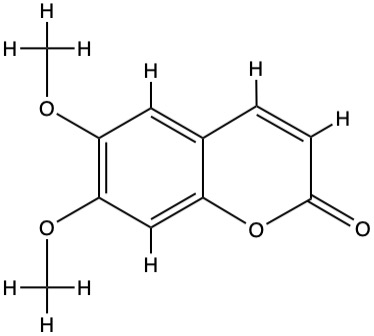 | Yinchen | CNKI | Simultaneous determination of fourteen constituents in Yinzhihuang granules by HPLC |
| wogonin | 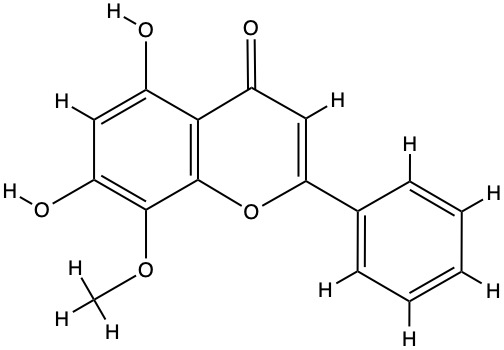 | Huangqin | CNKI | Simultaneous determination of fourteen constituents in Yinzhihuang granules by HPLC |
| oroxylin A | 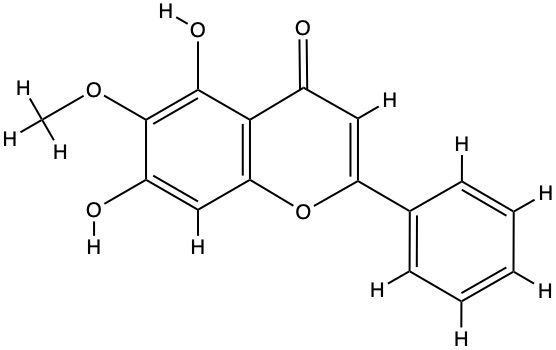 | Huangqin | CNKI | Simultaneous determination of fourteen constituents in Yinzhihuang granules by HPLC |
| Hyperoside | 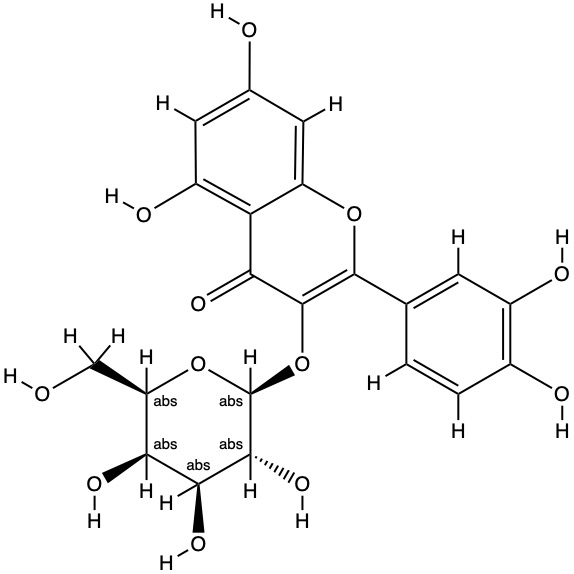 | Jinyinhua | CNKI | Simultaneous Determination of Four Components in Yinzhihuang Granules by High Performance Liquid Chromatography |
| Caffeic acid | 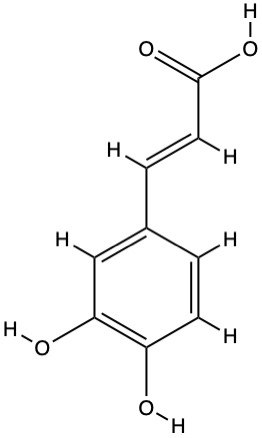 | Jinyinhua | PUBMED | Simultaneous Determination of 11 Components in Yinzhihuang Preparations and Their Constituent Herbs by High-Performance Liquid Chromatography with Diode Array Detector |
| geniposidic acid | 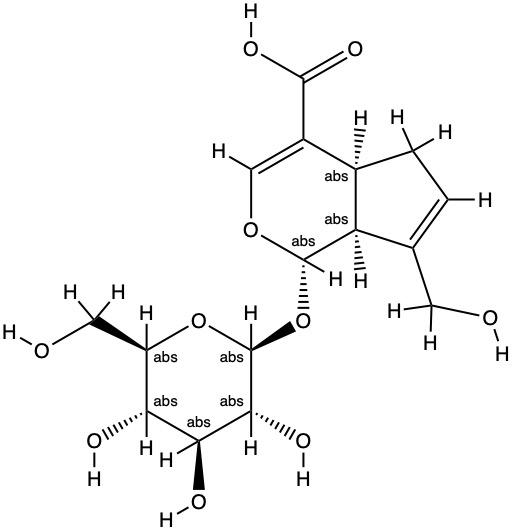 | Zhizi | PUBMED | Simultaneous Determination of 12 Components in Yinzhihuang Preparations and Their Constituent Herbs by High-Performance Liquid Chromatography with Diode Array Detector |
| luteoloside | 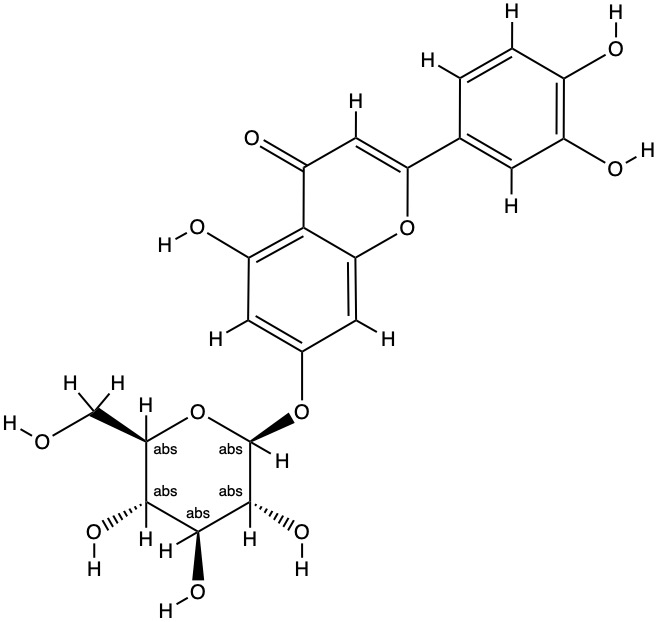 | Jinyinhua | PUBMED | Simultaneous Determination of 13 Components in Yinzhihuang Preparations and Their Constituent Herbs by High-Performance Liquid Chromatography with Diode Array Detector |
| luteolin | 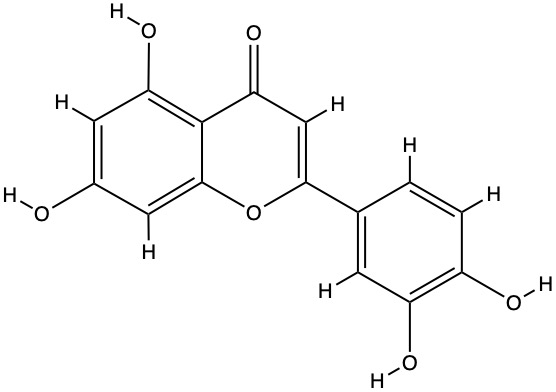 | Jinyinhua | PUBMED | Simultaneous Determination of 14 Components in Yinzhihuang Preparations and Their Constituent Herbs by High-Performance Liquid Chromatography with Diode Array Detector |

Supplementary Table S1: The information for the 25 compounds in YZHG.

| Target | Source |
| --- | --- |
| HMGCR | TTD |
| TLR7 | TTD |
| IFNA8 | TTD |
| IFNAR1 | TTD |
| TLR9 | TTD |
| DDX58 | TTD |
| NOD2 | TTD |
| HLA-DPA1 | pharmgkb |
| IFNA1 | disgenet |
| IFNG | disgenet |
| IFNA2 | disgenet |
| SLC10A1 | disgenet |
| IFNB1 | disgenet |
| HP | disgenet |
| IFNAR2 | disgenet |
| IL10RB | disgenet |
| IFNGR1 | disgenet |
| LOX | disgenet |
| LOXL2 | disgenet |
| HLA-DPB1 | disgenet |
| ALDH2 | disgenet |
| HLA-DQB2 | disgenet |
| PNPLA3 | disgenet |
| UBE2L3 | disgenet |
| GPT | disgenet |
| TP53 | disgenet |
| TNF | disgenet |
| IFNA13 | disgenet |
| PAEP | disgenet |
| AFP | disgenet |
| IFNL3 | disgenet |
| IL10 | disgenet |
| ALB | disgenet |
| IL6 | disgenet |
| CDKN2A | disgenet |
| HNF4A | disgenet |
| HLA-A | disgenet |
| GP1BA | disgenet |
| GGT1 | disgenet |
| TCF19 | disgenet |
| ITPR3 | disgenet |
| EHMT2 | disgenet |

Supplementary Table S2: The information for the 42 Hepatitis B targets.

| Category | Term | Count | PValue | Genes | FDR |
| --- | --- | --- | --- | --- | --- |
| GOTERM_MF_DIRECT | GO:0044212~transcription regulatory region DNA binding | 6 | 2.25E-07 | TNF, JUN, CREB1, TP53, BRCA1, STAT3 | 0.000243064 |
| GOTERM_BP_DIRECT | GO:0045893~positive regulation of transcription, DNA-templated | 7 | 6.38E-07 | TNF, JUN, CREB1, TP53, BRCA1, STAT3, CDK2 | 0.000916109 |
| GOTERM_BP_DIRECT | GO:0010628~positive regulation of gene expression | 6 | 6.45E-07 | TNF, TP53, CDK6, BRCA1, STAT3, TLR9 | 0.000925117 |
| GOTERM_BP_DIRECT | GO:0045944~positive regulation of transcription from RNA polymerase II promoter | 8 | 1.39E-06 | NOD2, TNF, JUN, CREB1, TP53, BRCA1, STAT3, TLR9 | 0.001994508 |

Supplementary Table S3: GO enrichment of potential target of YZHG for Hepatitis B (FDR < 0.01).

| Category | Term | Count | PValue | Genes | FDR |
| --- | --- | --- | --- | --- | --- |
| KEGG  PATHWAY | hsa05161:  Hepatitis B | 7 | 6.59E-08 | TNF, JUN, CREB1, TP53, CDK6, STAT3, CDK2 | 0.000075600 |
| KEGG  PATHWAY | hsa05203:Viral carcinogenesis | 6 | 1.50E-05 | JUN, CREB1, TP53, CDK6, STAT3, CDK2 | 0.017161555 |

Supplementary Table S4: KEGG enrichment of potential target of YZHG for Hepatitis B (FDR < 0.05).
